# Predictive Value of the Glasgow Prognostic Score for Prognosis in Patients with Hypopharyngeal Squamous Cell Carcinoma Treated with Curative Radiotherapy

**DOI:** 10.3390/jcm14145050

**Published:** 2025-07-16

**Authors:** Yuki Kasuga, Atsuto Katano, Subaru Sawayanagi, Masanari Minamitani, Yuki Saito, Koji Yamamura, Kenya Kobayashi, Hideomi Yamashita

**Affiliations:** 1 Department of Radiology, The University of Tokyo Hospital, 7-3-1 Hongo, Tokyo 113-0033, Japan; 2 Department of Otolaryngology—Head and Neck Surgery, The University of Tokyo Hospital, 7-3-1 Hongo, Tokyo 113-0033, Japan

**Keywords:** hypopharyngeal cancer, radiotherapy, Glasgow prognostic score, systemic inflammation, survival, biomarker

## Abstract

**Background/Objectives:** Hypopharyngeal squamous cell carcinoma (HPSCC) carries a poor prognosis, and reliable, inexpensive biomarkers are needed to refine risk-stratified treatment. The Glasgow Prognostic Score (GPS), integrating C-reactive protein and albumin, reflects systemic inflammation and nutritional status, but its prognostic utility in curative radiotherapy for HPSCC remains unclear. **Methods:** We retrospectively reviewed 98 consecutive patients with pathologically confirmed HPSCC who received definitive tomotherapy (70 Gy in 35 fractions) from June 2015 to February 2024 at a single tertiary center. Pretreatment GPS was classified as 0–2. Overall survival (OS) and progression-free survival (PFS) were assessed by Cox proportional hazards models, which evaluated associations between GPS and other clinical parameters. **Results:** Median age was 68 years (range 41–89); 92% were male. GPS distribution was 0 in 74 patients (76%), 1 in 18 (18%), and 2 in 6 (6%). After a median follow-up of 36.2 months, 3-year OS and PFS for the whole cohort were 78.7% and 51.7%, respectively. Patients with GPS 0 showed significantly higher 3-year OS than those with GPS 1–2 (83.6% vs. 62.2%; *p* = 0.023). On multivariate analysis, elevated GPS (1–2) remained an independent predictor of worse OS (hazard ratio [HR] 2.62, 95% CI 1.03–6.70; *p* = 0.044) alongside poor performance status and advanced stage. **Conclusions:** Pretreatment GPS independently stratifies overall survival in HPSCC patients undergoing curative radiotherapy, complementing established clinical factors. Because CRP and albumin are routinely available, GPS may assist in identifying high-risk patients who could benefit from intensified multidisciplinary treatment. Prospective multicenter studies are warranted to validate these findings.

## 1. Introduction

Hypopharyngeal squamous cell carcinoma (HPSCC) is a particularly aggressive form of head and neck cancer (HNC) with a poor prognosis [1]. HPSCC is among the most challenging HNCs in terms of prognosis, with a reported 5-year overall survival (OS) rate of only approximately 30–35% [2]. The complex anatomical structures of the hypopharynx, coupled with the typically advanced stage at diagnosis, contribute to the challenges in managing this malignancy [3]. Curative radiotherapy, often combined with chemotherapy, is the cornerstone treatment for patients who are not candidates for surgery [4,5,6]. However, predicting the treatment outcomes and overall prognosis remains a significant challenge in clinical practice.

In the search for reliable prognostic markers, systemic inflammation has been identified as a crucial factor influencing cancer progression and patient survival [7]. Numerous inflammatory markers have been recognized as prognostic predictors in patients with cancer. Among these, inflammatory cytokines are critical, playing key roles in cancer development, prognosis, and treatment [8]. Interleukins and tumor necrosis factors are particularly noteworthy. The prognostic significance of serum C-reactive protein (CRP) levels has been extensively researched in head and neck squamous cell carcinoma [9,10]. HPSCC is characterized by submucosal tumor spread that often extends beyond the macroscopically visible tumors [11]. This infiltrative growth pattern is frequently associated with localized immune responses at the invasive front, including the accumulation of tumor-infiltrating lymphocytes. These local immune interactions may, in turn, contribute to systemic inflammation, as indicated by elevated serum CRP levels [12]. Serum albumin level is also recognized as a marker reflecting malnutrition, impaired immune function, and chronic inflammation. It has been identified as an independent predictor of cancer-related mortality and treatment outcomes [13,14].

Based on these observations, the Glasgow Prognostic Score (GPS) was developed as an inflammation- and nutrition-based composite index, calculated using serum CRP and albumin levels [15]. GPS is simple, noninvasive, and easily applicable in routine clinical settings. It has been widely validated as an independent prognostic factor in various malignancies, including colorectal and esophageal cancers [16,17,18]. GPS has also been evaluated in cancers treated with definitive radiotherapy or chemoradiotherapy, showing prognostic value in esophageal squamous cell carcinoma and inoperable non-small cell lung cancer [19,20]. However, its prognostic role in HPSCC remains poorly defined, particularly in patients treated with curative radiotherapy. Given the high toxicity burden and treatment complexity associated with definitive radiotherapy, there is a clear need for simple and reliable tools to stratify patients by prognosis before treatment.

This retrospective study aimed to evaluate the association between pretreatment GPS and survival outcomes in patients with HPSCC treated with curative radiotherapy at a single institution. By validating the clinical utility of GPS in this setting, we seek to provide evidence to support its use as a practical prognostic marker that may help optimize treatment strategies and facilitate personalized care in HPSCC management.

## 2. Materials and Methods

This single-institution retrospective study was designed to evaluate the prognostic value of the GPS in patients diagnosed with HPSCC treated with curative radiotherapy. The study was approved by the Research Ethics Committee of the Faculty of Medicine of the University of Tokyo (No. 3372-7), and all patient data were anonymized to ensure confidentiality.

Patients included in this study had a pathologically confirmed diagnosis of HPSCC, were treated with curative-intent radiotherapy between June 2015 and February 2024, and had complete medical records, including pretreatment blood tests. Patients were excluded if they had previously undergone treatment for HNC, showed evidence of distant metastasis at the time of diagnosis, or had incomplete clinical or laboratory data. Data were collected from electronic medical records and included demographic information such as age and sex; clinical characteristics such as tumor stage and nodal status; treatment details such as the use of concurrent chemotherapy and upfront neck dissection; and follow-up information, including recurrence and survival status.

After completion of treatment, all patients were followed closely through a collaborative effort between our department and board-certified otolaryngologists. During the first year after treatment, follow-up visits were scheduled approximately every 1–2 months. In the second year, patients were seen every 2–3 months, and thereafter every 3–6 months based on clinical status. Each visit included a physical examination and flexible fiberoptic laryngopharyngoscopy. CT scans of the neck and chest were performed every 3–6 months to monitor for regional or distant metastases.

All patients were treated with intensity-modulated radiotherapy using tomotherapy (Accuray Inc., Sunnyvale, CA, USA), employing a three-level simultaneous integrated boost technique with curative intent. The total dose and fractionation schedule were determined to be 70 Gy in 35 fractions based on institutional protocols. All patients completed radiotherapy as planned, with a total dose of 70 Gy in 35 fractions. Computed tomography (CT) simulation for radiation therapy planning was performed with the patient immobilized using a thermoplastic mask. Gross tumor volume (GTV) included the primary tumor and enlarged lymph nodes. The high-risk clinical target volume (CTV 70 Gy) was defined as the GTV plus a 5–10 mm margin. The intermediate-risk volume (CTV 59.5 Gy) included prophylactic lymph node levels on the ipsilateral side of the involved lymph nodes, and the low-risk volume (CTV 54 Gy) included prophylactic lymph node levels on the contralateral side. These radiotherapy principles were based on international consensus recommendations regarding both primary tumor targeting and prophylactic lymph nodes [21,22]. The planning target volume was generated by adding a 3 mm margin to each CTV. The GPS was calculated based on pretreatment blood parameters, specifically CRP and albumin levels. A GPS of 0 was assigned when the CRP was ≤1.0 mg/dL and the albumin was ≥3.5 g/dL. A GPS of 1 was assigned when the CRP level was >1.0 mg/dL or the albumin level was <3.5 g/dL. A GPS of 2 was assigned when the CRP level was >1.0 mg/dL and the albumin level was <3.5 g/dL.

All statistical analyses were performed using R software (version 4.1.2; R Foundation for Statistical Computing, Vienna, Austria). Continuous variables are summarized as median ranges, depending on the data distribution. Categorical variables are presented as frequencies and percentages. OS was defined as the time from the start of radiotherapy for HPSCC to death from any cause or the date of the last follow-up. Progression-free survival (PFS) was defined as the time from the initiation of radiotherapy for HPSCC to the first occurrence of disease progression or death from any cause, whichever occurred first. Survival analyses were conducted using the Kaplan–Meier method, and survival curves were compared using the log-rank test. Multivariate analysis was performed using a Cox proportional hazards model to identify independent prognostic factors. The following clinical variables were included simultaneously in a multivariate model: age, sex, performance status, tumor subsite, clinical stage, chemotherapy, upfront neck dissection, and Glasgow Prognostic Score. Hazard ratios (HRs), 95% confidence intervals (CIs), and *p*-values were calculated. Variables were selected based on clinical relevance rather than univariate significance, to control for potential confounders. Statistical significance was defined as a *p*-value < 0.05.

## 3. Results

This study included 98 patients with histologically verified HPSCC who underwent definitive radiotherapy, retrospectively analyzed (Table 1).

The median patient age was 68 years (range, 41–89 years). The cohort consisted of 90 men and 8 women. The Eastern Cooperative Oncology Group (ECOG) performance status was 0 in 81 patients and 1 in 17. The primary tumor was located in the pyriform sinus, posterior pharyngeal wall, and post-cricoid region in 78, 10, and 10 patients, respectively. The clinical stage was I in 16 patients, II in 34, III in 14, and IVA/IVB in 34. Fifty-two patients received chemotherapy, and eleven underwent upfront neck dissection prior to radiotherapy. Regarding chemotherapy, the majority of patients (49 out of 52) received concurrent cisplatin (CDDP) during radiotherapy using triweekly regimens. Among these, the most common protocol was a cumulative dose of 240 mg/m^2^, typically administered in three cycles on days 1, 22, and 43 of radiotherapy. Three others received alternative systemic therapy: one patient was treated with weekly docetaxel, one with cetuximab, and one received induction chemotherapy with the DCF regimen (docetaxel, cisplatin, and 5-FU) prior to radiotherapy. Upfront neck dissection was performed in 11 patients as part of a planned treatment strategy, based on multidisciplinary tumor board discussions. Indications included radiological suspicion of extranodal extension or bulky nodal disease [23]. Patients were stratified based on their GPS, with 74 patients categorized as having a GPS of 0, 18 as having a GPS of 1, and 6 as having a GPS of 2. Pretreatment liver function tests were available for 96, 97, and 86 patients for aspartate aminotransferase (AST), alanine aminotransferase (ALT), and total bilirubin (T-Bil), respectively. The median AST level was 23 U/L (range: 11–105 U/L), with 18 patients exceeding the institutional upper normal limit of 30 U/L. The median ALT level was 15 U/L (range: 4–78 U/L), with 7 patients above the normal threshold of 30 U/L. The median T-Bil level was 0.7 mg/dL (range: 0.2–1.8 mg/dL), and three patients had elevated levels above 1.5 mg/dL.

The median follow-up period was 36.2 months (range: 3.5–98.2 months). At 3 years, the OS rate was 78.7% (95% CI: 68.2–86.1) in the entire cohort, and the median OS was not reached (lower bound: 71.4 months). At 3 years, the PFS rate was 51.7% (95% CI: 40.4–61.8) in the entire cohort, with a median PFS of 40.2 months (95% CI: 23.0–NA).

**Figure 1:**
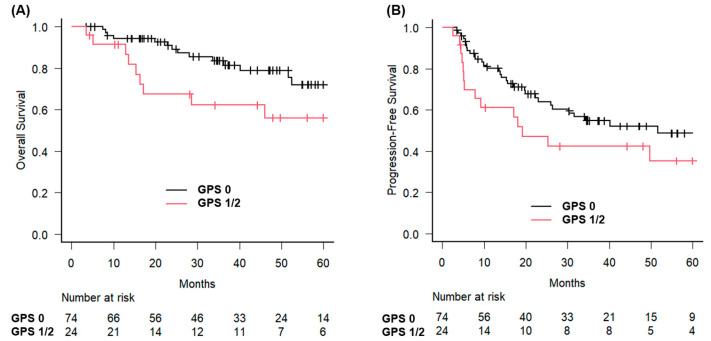
Overall and progression-free survival stratified by Glasgow Prognostic Score in patients with hypopharyngeal squamous cell carcinoma treated with curative radiotherapy. Kaplan–Meier survival curves illustrating (**A**) overall survival and (**B**) progression-free survival stratified by the Glasgow Prognostic Score (GPS) in patients with hypopharyngeal squamous cell carcinoma who received curative radiotherapy. The numbers of at-risk patients at each time point are shown in the graphs below. Censoring events are indicated by tick marks on the survival curves.

The 3-year OS rates were analyzed using the GPS. For patients with GPS 0, the 3-year OS rate was 83.6% (95% CI: 71.5–90.9%), and the median OS was not reached. In contrast, patients with GPS 1 or 2 had a 3-year OS rate of 62.2% (95% CI: 38.3–79.1%), and the median OS was 71.4 months (95% CI: 16.3 months–not estimable) (Figure 1).

**Figure 2:**
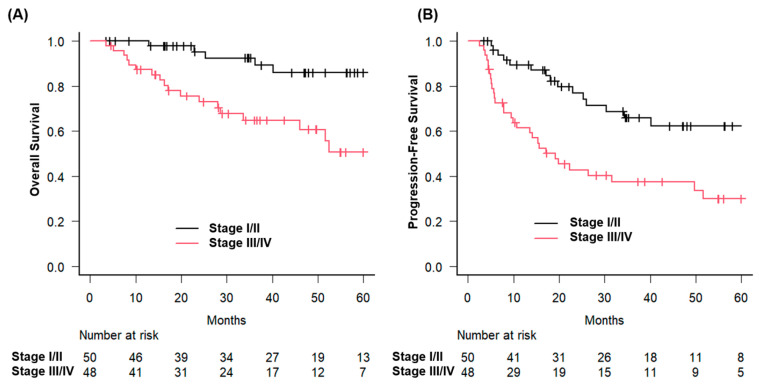
Overall and progression-free survival stratified by clinical stage in patients with hypopharyngeal squamous cell carcinoma treated with curative radiotherapy. Kaplan–Meier survival curves illustrating (**A**) overall survival (OS) and (**B**) progression-free survival (PFS) according to clinical stage in patients with hypopharyngeal squamous cell carcinoma treated with curative radiotherapy. Patients were categorized into early-stage (stage I/II, black line) and advanced-stage (stage III/IV, red line) groups. OS and PFS were significantly longer in the early-stage group than in the advanced-stage group.

The difference in OS between the groups was statistically significant (*p* = 0.023). Similarly, patients with early-stage disease had a 3-year OS of 92.5%, compared with 64.7% for those with advanced-stage disease (*p* = 0.0021) (Figure 2).

**Figure 3:**
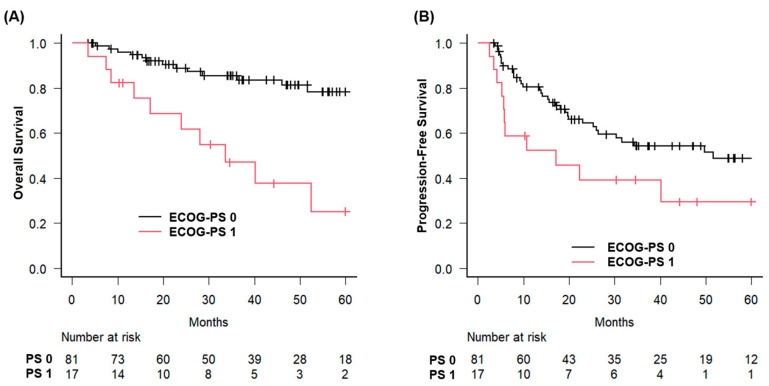
Overall and progression-free survival stratified by performance status in patients with hypopharyngeal squamous cell carcinoma treated with curative radiotherapy. Kaplan–Meier survival curves illustrating (**A**) overall survival (OS) and (**B**) progression-free survival (PFS) according to the Eastern Cooperative Oncology Group performance status (ECOG-PS) in patients with hypopharyngeal squamous cell carcinoma treated with curative radiotherapy. Patients were stratified into good performance status (ECOG-PS 0, black line) and poor performance status (ECOG-PS 1, red line) groups. OS and PFS were significantly better in patients with an ECOG-PS of 0 than in those with an ECOG-PS of 1.

In addition, patients with ECOG performance status 0 had a 3-year survival rate of 85.6% compared with 47.1% for those with status 1 (*p* < 0.001) (Figure 3).

In the univariate analysis for OS, an elevated GPS (0 vs. 1 or 2) was significantly associated with worse OS, with a hazard ratio (HR) of 2.485 (95% CI: 1.103–5.600; *p* = 0.028), which remained significant in the multivariate analysis (HR: 2.622; 95% CI: 1.027–6.697; *p* = 0.044) (Table 2).

Regarding other factors, univariate analysis showed that performance status (HR: 4.826, 95% CI: 2.105–11.063, *p* < 0.001) and clinical stage (HR: 3.863, 95% CI: 1.529–9.764, *p* = 0.004) were significantly associated with poorer prognosis. In multivariate analysis, performance status (HR: 6.034, 95% CI: 2.292–15.890, *p* < 0.001), clinical stage (HR: 21.840, 95% CI: 4.879–97.810, *p* < 0.001), and chemotherapy (HR: 0.091, 95% CI: 0.020–0.411, *p* = 0.002) remained independently associated with prognosis.

In the Cox proportional hazards analysis for PFS, GPS (0 vs. 1 or 2) was not statistically significant in univariate (HR: 1.687; 95% CI: 0.912–3.121; *p* = 0.096) or multivariate analysis (HR, 1.415; 95% CI, 0.718–2.787; *p* = 0.316), although it showed a trend toward a worse prognosis (Table 3).

Clinical stage was an independent predictor of PFS in univariate (HR: 2.525, *p* = 0.002) and multivariate analyses (HR: 4.627, *p* = 0.001). Chemotherapy demonstrated a significant protective effect in the multivariate analysis (HR: 0.330; 95% CI: 0.126–0.860; *p* = 0.023) despite being non-significant in univariate analysis.

## 4. Discussion

This study evaluated the prognostic value of the GPS in patients with HPSCC treated with curative radiotherapy. Our findings suggest that the GPS provides additional prognostic information that complements conventional clinical factors. Patients with a higher GPS (1 or 2) had a significantly worse OS than those with a GPS of 0. These findings are consistent with those of previous studies across various cancers, where an elevated GPS was associated with poor prognosis, reflecting the influence of systemic inflammation and malnutrition. The GPS is based on a simple combination of CRP and albumin levels, serving as a marker of systemic inflammation and nutritional status, respectively. In recent years, revised versions of the GPS have been proposed. McMillan reported that the modified Glasgow Prognostic Score (mGPS) has been validated as an independent prognostic indicator across various cancers in over 60 studies [15]. Moreover, the high-sensitivity modified Glasgow Prognostic Score (HS-mGPS) has been proposed to provide a superior prognostic value compared with the mGPS [24].

In our cohort, 47% of patients did not receive concurrent chemotherapy. This was largely due to the inclusion of early-stage cases (Stages I–II), for whom radiotherapy alone is often considered appropriate. In addition, chemotherapy was omitted in some patients due to age, comorbidities, or patient preference. Although all patients had good performance status, treatment decisions were made based on a comprehensive clinical assessment, including factors not fully captured by PS alone.

Recent studies have demonstrated that the GPS and its modifications, such as the mGPS and HS-mGPS, can serve as significant prognostic indicators for radiotherapy. In patients with muscle-invasive bladder cancer undergoing radiotherapy, pretreatment mGPS has been shown to predict OS, with 2-year OS rates of 85.1% for mGPS 0, 71.3% for mGPS 1, and 60.9% for mGPS 2 (*p* = 0.003) [25]. In a retrospective study involving 207 patients with non-small cell lung cancer treated with stereotactic body radiation therapy, the mGPS was found to be an independent predictor of prognosis [26]. Patients with a high mGPS (1–2) exhibited significantly worse outcomes, including lower OS (median OS: 33.3 vs. 64.5 months; *p* = 0.003) and PFS (median PFS: 23.8 vs. 39 months; *p* = 0.008). Ishikawa et al. reported that in a cohort of 180 patients undergoing whole-brain radiotherapy, a pretreatment GPS ≥ 1 was significantly associated with worse survival outcomes [27] (median survival time: 6.1 months), even after adjusting for established prognostic factors such as Karnofsky performance status and Graded Prognostic Assessment.

HPSCC, along with other head and neck cancers (HNC), is associated with a high risk of malnutrition and systemic inflammation due to tumor location, treatment-related toxicities, and impaired oral intake [28,29,30]. Nutritional status can significantly affect treatment tolerance and clinical outcomes. The United Kingdom National Multidisciplinary Guidelines emphasize the importance of nutritional support in managing HNC, noting that many patients are already malnourished at diagnosis and most require nutritional intervention during treatment [31]. At our institution, structured nutritional support is routinely considered, particularly for patients receiving concurrent chemoradiotherapy. As a general policy, prophylactic percutaneous endoscopic gastrostomy PEG placement is performed before the initiation of chemoradiotherapy. However, due to the retrospective design of our study, detailed data on the proportion of patients who received PEG or other forms of enteral nutrition were not consistently available. In this context, the pretreatment GPS may serve as a practical surrogate indicator reflecting the underlying nutritional and inflammatory status in patients with HPSCC.

Our findings demonstrated that a higher GPS, based on pretreatment CRP and albumin levels, was significantly associated with poorer survival in patients with HPSCC. While GPS is a practical and integrative biomarker reflecting both systemic inflammation and nutritional status, other indices such as the Prognostic Nutritional Index (PNI) and the neutrophil-to-lymphocyte ratio (NLR) have also been shown to predict outcomes in HNC. For example, a meta-analysis by Luan et al. confirmed that a low PNI was significantly associated with worse OS, and PFS, and elevated NLR has similarly been linked to inferior survival in both general HNC populations and hypopharyngeal cancer specifically [32]. Justesen et al. performed a retrospective cohort study of 1370 patients with oropharyngeal squamous cell carcinoma and found that higher pretreatment NLR was independently associated with worse OS and recurrence-free survival [33]. In Kuo et al.’s retrospective study of 120 patients with hypopharyngeal cancer treated with definitive chemoradiotherapy, high pretreatment NLR (≥4) was significantly associated with advanced tumor stage, poor treatment response, and worse PFS and OS [34]. These findings suggest that multiple pretreatment biomarkers may have complementary prognostic value, and further studies are needed to compare their relative and combined utility.

While the present study focused on pretreatment risk stratification, longitudinal nutritional management throughout the course of treatment may also play a critical role in improving patient outcomes. Previous studies have demonstrated that structured nutritional interventions, such as medical nutrition therapy and individualized counseling, can reduce weight loss, preserve body composition, and enhance both treatment tolerance and quality of life [35,36]. Notably, Molnár et al. reported that extended durations of nutritional support were associated with significantly improved overall survival [37]. These findings highlight the importance of not only assessing pretreatment nutritional status but also providing continuous, longitudinal nutritional care as an integral part of comprehensive cancer management.

This study had several limitations. This retrospective design introduced potential biases and restricted our ability to establish causality. Additionally, as this was a single-institution study, the generalizability of our findings may be limited. The sample size, which was sufficient to detect significant associations, may have affected the broader applicability of our results. Furthermore, we were unable to assess patients’ nutritional status or sarcopenia using standardized tools such as NRS-2002 and SARC-F, as these assessments were not routinely performed in our cohort. Future multicenter studies with larger cohorts are necessary to validate our findings, assess the generalizability of the GPS in predicting HPSCC outcomes, and incorporate validated screening tools to enhance the evaluation of patient-related prognostic factors.

## 5. Conclusions

Our study supports the use of the GPS as a promising prognostic tool for patients with HPSCC who are undergoing curative radiotherapy. Higher GPS values are associated with worse overall outcomes, independent of traditional clinical factors. The simplicity and cost-effectiveness of the GPS make it a practical addition to clinical practice, aiding in risk stratification and treatment planning. Further research is needed to validate these results and explore the potential integration of the GPS into clinical practice guidelines for HPSCC.

## Figures and Tables

**Table 1:** Baseline clinical characteristics of patients with hypopharyngeal cancer treated with definitive radiotherapy.

Variables	Number (Percentage)
Age: median (range)	68 (41–89)
Sex	
Male	90 (92%)
Female	8 (8%)
ECOG performance status	
0	81 (83%)
1	17 (17%)
Tumor subsite	
Pyriform sinus	78 (80%)
Posterior pharyngeal wall	10 (10%)
Post-cricoid region	10 (10%)
Clinical stage	
I	16 (16%)
II	34 (35%)
III	14 (14%)
IVA/IVB	34 (35%)
Chemotherapy	
No	46 (47%)
Yes	52 (53%)
Upfront neck dissection	
No	87 (89%)
Yes	11 (11%)
Smoking status	
Never smoked	11 (11%)
Former smoker	63 (64%)
Current smoker	25 (25%)
Brinkman Index	
0	11 (11%)
1–399	38 (39%)
400–799	32 (33%)
≥800 or more	17 (17%)
Glasgow Prognostic Score	
0	74 (76%)
1	18 (18%)
2	6 (6%)

**Table 2:** Univariate and multivariate Cox proportional hazards analysis for overall survival.

		Univariate			Multivariate		
Covariables		Hazard Ratio	95% CI	*p* Value	Hazard Ratio	95% CI	*p* Value
Age	≤70 vs. >70 years	1.007	0.441–2.303	0.986	0.485	0.164–1.437	0.192
Sex	Male vs. female	1.401	0.327–5.999	0.649	3.620	0.742–17.670	0.112
Performance status	0 vs. 1	4.826	2.105–11.063	<0.001	6.034	2.292–15.890	<0.001
Tumor subsite	Pyriform sinus vs. others	0.823	0.281–2.411	0.723	1.366	0.420–4.441	0.604
Clinical stage	I, II vs. III, IVA/B	3.863	1.529–9.764	0.004	21.840	4.879–97.810	<0.001
Chemotherapy	No vs. yes	1.340	0.594–3.022	0.481	0.091	0.020–0.411	0.002
Upfront neck dissection	No vs. yes	1.266	0.377–4.254	0.703	0.358	0.092–1.393	0.138
GPS	0 vs. 1, 2	2.485	1.103–5.600	0.028	2.622	1.027–6.697	0.044

**Table 3:** Univariate and multivariate Cox proportional hazards analysis for progression-free survival.

		Univariate			Multivariate		
Covariables		Hazard Ratio	95% CI	*p* Value	Hazard Ratio	95% CI	*p* Value
Age	≤70 vs. >70 years	0.686	0.371–1.269	0.230	0.529	0.252–1.109	0.092
Sex	Male vs. female	1.586	0.623–4.038	0.333	1.704	0.629–4.618	0.295
Performance status	0 vs. 1	2.000	1.013–3.95	0.046	1.917	0.906–4.056	0.089
Tumor subsite	Pyriform sinus vs. others	0.833	0.403–1.726	0.624	0.860	0.396–1.866	0.702
Clinical stage	I, II vs. III, IVA/B	2.525	1.387–4.597	0.002	4.627	1.805–11.860	0.001
Chemotherapy	No vs. yes	1.331	0.748–2.368	0.331	0.330	0.126–0.860	0.023
Upfront neck dissection	No vs. yes	2.133	0.99–4.594	0.053	0.972	0.412–2.289	0.947
GPS	0 vs. 1, 2	1.687	0.912–3.121	0.096	1.415	0.718–2.787	0.316

## Data Availability

Data supporting the findings of this study are available from the corresponding author on reasonable request.
